# Personalized Route Planning System Based on Driver Preference

**DOI:** 10.3390/s22010011

**Published:** 2021-12-21

**Authors:** Ren Wang, Mengchu Zhou, Kaizhou Gao, Ahmed Alabdulwahab, Muhyaddin J. Rawa

**Affiliations:** 1 Institute of Systems Engineering, Macau University of Science and Technology, Macau 999078, China; ren_112358@163.com (R.W.); gaokaizh@aliyun.com (K.G.); 2 Center of Research Excellence in Renewable Energy and Power Systems, Department of Electrical and Computer Engineering, Faculty of Engineering, King Abdulaziz University, Jeddah 21481, Saudi Arabia; aabdulwhab@kau.edu.sa (A.A.); mrawa@kau.edu.sa (M.J.R.)

**Keywords:** optimization, personalization, preference, route planning, crowd sensing, global positioning system, geographic information system

## Abstract

At present, most popular route navigation systems only use a few sensed or measured attributes to recommend a route. Yet the optimal route considered by drivers needs be based on multiple objectives and multiple attributes. As a result, these existing systems based on a single or few attributes may fail to meet such drivers’ needs. This work proposes a driver preference-based route planning (DPRP) model. It can recommend an optimal route by considering driver preference. We collect drivers’ preferences, and then provide a set of routes for their choice when they need. Next, we present an integrated algorithm to solve DPRP, which speeds up the search process for recommending the best routes. Its computation cost can be reduced by simplifying a road network and removing invalid sub-routes. Experimental results demonstrate its effectiveness.

## 1. Introduction

Navigation systems have become increasingly popular, especially the navigation software in mobile phones, from which we can obtain navigation services at no or low cost. People have various requirements for services, and may be unsatisfied with the shortest or fastest route as offered by most of today’s navigation systems in use. Mainstream navigation systems usually focus on distance, driving time, the number of traffic lights, and tolls. However, in reality there are several other attributes that affect driver choice and experience, such as driving safety, difficulty and scenery. A navigation system receives a higher satisfaction score from drivers when the attributes affecting driver choices are considered more comprehensively. Doing so requires us to consider more attributes and driver preferences in the process of route planning [1]. On the other hand, due to individual differences, each driver’s preferences are different, and the recommended routes from a navigation system are expected to be different, which is beneficial in reducing the congestion often seen in today’s transportation networks.

With the development of relevant technologies such as Internet of Things [2,3], road information can be sensed and obtained more quickly and accurately, and the real-time road condition can thus be provided to drivers [4,5]. For example, through a Global Positioning System (GPS) and Geographic Information System (GIS), a user’s location, the length and width of a road and the other basic attributes can be quickly acquired. Real-time traffic flow and congestion can be obtained through a surveillance and control system and crowd sensing [6,7,8,9,10,11]. Some models are getting better, such as a travel time prediction model and congestion estimation model given historical and real-time sensed data [12,13,14,15]. Therefore, it is technically possible to consider more attributes and driver preferences in a next-generation navigation system. 

Pang, et al. [6] establish a framework of route planning based on driver preference. They use fuzzy-neural methods to represent the correlation between route attributes and driver route selection, and optimize the recommended route by training a fuzzy-neural network. Park, et al. [1] suggest a decision tree learning algorithm and a model to represent driver choice behavior with respect to driver preferences. The selection rules are adaptively updated when the proposed model finds a difference between the predicted selection and the actual selection made by a driver. Nadi and Delavar [16] present a generic model that combines a pairwise comparison method and quantifier-guided ordered weighted average (OWA) aggregation operators to form a personalized route planning model. The model calculates the impedance of each link based on routing attributes, attributes importance, and selected decision strategies, and considers driver preferences. Pahlavani and Delavar [17] design a neuro-fuzzy toolbox to learn driver preferences in multi-standard path selection. The local linear neuro-fuzzy models (LLNFM) and adaptive neuro-fuzzy inference systems (ANFIS) are proposed and compared. Abdelhamid, et al. [12] propose a dynamic route guidance system that focuses on a driver’s satisfaction and considers driver behavior in a route guidance process to boost the safety levels on roads. Teng, et al. [18] propose a dynamic route search method considering individual driver preferences. The fuzzy-analytic hierarchy process (AHP) and D-star are used to integrate multiple preferences and realize route searches. Zheng, et al. [14] present a multi-objective path planning model and use the analytic hierarchy process (AHP) and gray relational analysis to obtain the relative properties of each sub- objective according to driver preference. Ntakolia and Iakovidis [19] propose a swarm intelligence graph-based pathfinding algorithm (SIGPA) to solve the multi-objective path model of electric vehicles.

There are other related studies on personalized route planning [20,21,22,23]. Papinski, et al. [24] use the interview and questionnaire methods to obtain the sort of attributes that drivers care about. Fujii, et al. [25] study the readability of a user interface. Kashevnik, et al. [26] propose an approach to a driver decision support system that is based on an Internet-of-Transportation-Things concept. This approach collects and processes information through smart-phones mounted in vehicle windshield, and uses the information to provide personalized recommendations for a driver.

The core function of the proposed driver preference-based route planning (DPRP) is the planning of a route. Its main difference from traditional navigation systems is that drivers’ personal preference is more comprehensively considered, and their selection of recommended routes is collected. Here, we use a road network (nodes and edges) to represent a map, the junctions are represented by nodes, and the segments are represented by edges. We use a weight to measure the importance of attributes such that important attributes are given high weight values. We use the attribute values of a road to calculate the “road resistance” in order to accurately reflect its characteristics. Here “road resistance” is like “route score”. However, a smaller value of road resistance indicates that a route is easier to pass. Road resistance is used to rank alternate routes and DPRP recommends a route to drivers with the lowest value of road resistance. The basic attribute values are collected and normalized. For a given origin–destination (*O*-*D*) pair, there may be too many routes to use. Nonetheless, routes that are too long are meaningless to a driver in general. It is thus necessary to use distance control to restrict the number of routes to be considered.

The performance of DPRP can be evaluated in terms of the degree to which the model’s recommended route fits a driver’s idea. Therefore, it is very important whether the weight is appropriate or not. We can directly ask drivers to input their preference, which is the most effective method to obtain weight [27]. However, drivers may not accurately express what they want when setting their preference. In order to reduce this error and increase the speed of calculation, we use big data and a questionnaire method to obtain a driver’s preference when they get confused about the importance of attributes. 

Drivers’ requirements for recommended routes are increasing. They are no longer satisfied with just the shortest or fastest route. Different drivers have different requirements to meet their different purposes. For example, some female or elderly drivers may prefer fewer cars and wider roads; while travelers want to drive on a more scenic route. When recommending a route, it is important to consider drivers’ preference, which can increase the utilization rate of the road network and alleviate congestion to a certain extent. Although there have been some studies on driver preferences, the impact attributes considered by most existing studies are not comprehensive enough, and these models have few experiments based on actual road networks. This is the motivation of our study.

The main contributions of this paper can be summarized as follows:

(1) We propose a new route recommendation model that comprehensively considers the attributes that affect a driver’s choice for a route; and
(2) We propose an approach to reduce a solution set in which a DPRP model is solvable within an acceptable time range. An algorithm to solve a DPRP model is proposed. Four experiments are performed to verify its performance.

The remainder of this paper is organized as follows. The mathematical model of road resistance for alternative routes is formulated in Section 2. Section 3 proposes three algorithms to solve the model. Section 4 presents the experimental results. Finally, Section 5 concludes this paper.

## 2. Problem Description and Formulation

In this section, we first elaborate on the DPRP problem we care about and define the parameters. Finally, we describe its solution.

### 2.1. Problem Description

Drivers may choose a route based on a number of attributes, and it is not always satisfactory to recommend a route according to only one or two attributes, e.g., minimum travel time. Compared with traditional navigation systems and related research, we consider more attributes affecting a driver’s choice, and make a recommended route more in line with the true requirements of a driver. 

In order to truly reflect the road conditions in a calculation process, the processed physical quantity is directly used as road resistance, and calculate the route with the lowest value of road resistance and recommend it to a driver. Here, we need to define “segment” and “route”. A segment represents a road between two adjacent intersections and a route means a sequence of segments between an origin and destination.

The main notations in this paper are summarized in Table 1.

### 2.2. Attributes of Segments and Routes

(1) Definitions of attributes

Drivers’ choices may be influenced by many attributes, including scenery, radius of curvature, the number of lanes, lane width, distance, congestion, traffic flow, the number of pedestrians and bicycles, congestion rate, cost of time, fuel consumption, toll-fee, traffic lights, intersections, the number of turns and the separation of motor vehicles, and non-motor vehicles. They are defined and explained to facilitate understanding as follows:

- Scenery (*p_i,1_*): its value ranges from zero to ten where 10 denotes the best scenery. The higher, the better.
- Radius of curvature (*p_i,2_*): It indicates the degree of road curvature. The larger, the better.
- Congestion (*p_i,6_*): characterized by traffic queue length that indicates the degree of congestion.
- Traffic flow (*p_i,7_*): the number of vehicles in a segment over its length.
- The number of pedestrians and bicycles (*p_i,8_*): the number of pedestrians and bicycles in a segment over its length.
- Congestion rate (*p_i,9_*): the historical situation of the traffic jam at a fixed time is recorded, and the probability of congestion is computed during that period.
- The separation of motor vehicles and non-motor vehicles (*p_i,10_*): represented by a binary variable. When a segment does not separate motor vehicles and non-motor vehicles, *p_i,10_* = 1; otherwise, *p_i,10_* = 0.

The attributes considered are summarized in Table 2. Some of them need to be computed based on historical data.

(2) Types of attributes

Those attributes that drivers would like to be as large as possible in an ideal route are called positive ones. Those they prefer to be as small as possible are called negative ones. We have the following classified attributes:

- Positive ones: scenery *(p_i,1_*), radius of curvature (*p_i,2_*), the number of lanes (*p_i,3_*) and lane width (*p_i,4_*).
- Negative ones: distance (*p_i,5_*), congestion (*p_i,6_*), traffic flow (*p_i,7_*), the number of pedestrians and bicycles (*p_i,8_*), congestion rate (*p_i,9_*), the separation of motor vehicles and non-motor vehicles (*p_i,10_*), cost of time (*r_m,1_*), fuel consumption (*r_m,2_*), toll fee (*r_m,3_*), the number of traffic lights (*r_m,4_*), the number of intersections (*r_m,5_*) and the number of turns (*r_m,6_*).

In this paper, a segment is taken as a basic entity, and the values of each attribute are calculated. But some attributes (such as the number of traffic lights) must be based on a route. Therefore, they are considered under *O-D*, which are called route attributes. They are: cost of time (*r_m,1_*), fuel consumption (*r_m,2_*), toll fee (*r_m,3_*), the number of traffic lights (*r_m,4_*), the number of intersections (*r_m,5_*) and the number of turns (*r_m,6_*). Other attributes are called segment attributes.

(3) Processing of attributes

Since route attributes need to be considered based on *O*-*D*, we have to discuss them separately. Two attributes sets are represented as two matrices, i.e., P for segment attributes and *R* for route attributes. *P* is an *I* × *J* matrix, where there are *I* segments that have *J* attributes; *R* is an *M* × *N* matrix, where there are *M* routes each of which has N attributes, i.e.,

*P* = [*p*_*i*,*j*_] _*I×J*_ (1)

*R* = [*r*_*m,n*_] _*M×N*_ (2)

Note that in order to reduce the ambiguity of the attributes and avoid subjective influences, only the scores of the scenery are given by experts and user review scores, and the rest of the attributes are all derived from the objective values based on physics, historical data and/or mathematical models.

In the second step, the matrices are normalized such that all involved elements are treated as being unit-free, which allows for easier comparisons across attributes.

(3) $$q_{i,j}=\{\begin{array}{ll} \frac{{\overset{ˇ}{p}}_{j}\;}{p_{i,j}} & \forall j,\;p_{j}\;\mathrm{is}\;a\;\mathrm{positive}\;\mathrm{attribute}\\ \frac{p_{i,j}}{{\hat{p}}_{j}} & \forall j,\;p_{j}\;\mathrm{is}\;a\;\mathrm{negative}\;\mathrm{attribute} \end{array}$$

(4) $$\lambda_{m,n}=\{\begin{array}{ll} \frac{{\overset{ˇ}{r}}_{n}\;}{r_{m,n}} & \forall n,\;r_{n}\;\mathrm{is}\;a\;\mathrm{positive}\;\mathrm{attribute}\\ \frac{r_{m,n}}{{\hat{r}}_{n}} & \forall n,\;r_{n}\;\mathrm{is}\;a\;\mathrm{negative}\;\mathrm{attribute} \end{array}$$

(5) $${\tilde{p}}_{i,j}=\frac{q_{i,j}\;-\;{\overset{ˇ}{q}}_{j}}{{\hat{q}}_{j}\;-\;{\overset{ˇ}{q}}_{j}}$$

(6) $${\tilde{r}}_{m,n}=\frac{\lambda_{m,n}-{\overset{ˇ}{\lambda }}_{n}}{{\hat{\lambda }}_{n}-{\overset{ˇ}{\lambda }}_{n}}$$

After applying (3)–(6) to the matrices *P* and *D*, two normalized matrices $\tilde{P}$ and $\tilde{R}$ are obtained where ${\tilde{p}}_{i,j}$ contain the normalized attribute value for segment *i* and attribute *j*; ${\tilde{r}}_{m,n}$ contain the normalized attribute value for route *m* and attribute *n*.

(7) $$\tilde{P}={\left[{\tilde{p}}_{i,j}\right]}_{I\times J}$$

(8) $$\tilde{R}={\left[{\tilde{r}}_{m,n}\right]}_{M\times N}$$

### 2.3. Weight

In some situations, drivers may have specific preferences. This can happen with any trip planning. Drivers need to consider the importance of the attributes that influence their choice in different situations. Among many attributes, we need to determine which are more important to recommend a reasonable route for drivers. For example, if a driver is very concerned with congestion, and is not concerned about the scenery, then the weight of the congestion can be increased and the scenery’s weight can be reduced. The recommended routes should give more weight to “congestion” and less or zero weights to “scenery”.

(1) Big data-based setting

With the widespread use of the Internet and the popularity of mobile devices equipped with GPS (such as vehicles GPS, cellphones and wearable devices), every time a user using a computer or mobile device leaves the arrays of footprints, which accumulate a huge collection of user data, including social media data, search records and driving trajectories. The technology of the recommendation system based on big data considering preferences has been effectively used in the area of shopping, medical treatment, tourism and other fields. There are also some studies about route planning. A series of data such as the information released by users on social media, shopping search records and historical travel trajectories can be analyzed to give a weight in line with driver’s preference [28,29,30,31,32,33].

DPRP records weight generated by each use and collects evaluation reports of drivers after use. Such data can be used to adjust weight accordingly.

(2) Driver setting

There is a subset of drivers who are willing to take additional time to provide their preferences. In this case, we require drivers to select the importance of attributes when using a DPRP model to obtain driver preferences directly. Options are shown in Table 3. Here, we take scenery as an example, and other attributes are the same as it. According to the purpose of a trip and personal preference, a driver chooses a corresponding option. If drivers are uncertain about setting certain attributes, they can choose “Default” by which our model automatically sets those attributes, e.g., to allow the users to have partial setting to start their trip. Note that in some cases, different preferences lead to the same route. Each option represents a weight value that indicates the importance of the attribute. The values of each option are as follows:
“Most important”1.0“Very important”0.8“Important”0.6“Slightly important”0.4“Least important”0.2“No importance at all”0.0

A driver can directly choose the importance of the attributes, but sometimes people cannot fully express what they want. In this case, DPRP helps drivers make choices when they are unsure. If there is no option that can express drivers’ expectations, they can ask DPRP for help, which is expected to give them an option that matches their preferences. Each time a driver completes a preference setting, a questionnaire is completed. DPRP collects and groups them. The database is continuously expanded as the number of users increases, and then more suitable routes can be provided.

### 2.4. Driver Preference-Based Route Planning (DPRP) Model

DPRP takes a segment as a basic entity, which can be used to characterize road conditions fundamentally and conform to the actual road condition as much as possible. Calculated values mainly come from the processed attribute values. Only the scenery cannot be objectively evaluated, and as experts and user review scores increase, the impact of subjective evaluations decreases. In order to accurately recommend a preferred route for a driver, it is necessary to classify the attributes that we consider.

(1) Segment resistance

The segment attributes include scenery (*p_i,1_*), radius of curvature (*p_i,2_*), the number of lanes (*p_i,3_*), lane width (*p_i,4_*), distance (*p_i,5_*), congestion (*p_i,6_*), traffic flow (*p_i,7_*), the number of pedestrians and bicycles (*p_i,8_*), and rate of congestion (*p_i,9_*) and the separation of motor vehicles and non-motor vehicles (*p_i,10_*). They are the basic physical properties of a segment and do not change with a route. The segment resistance is calculated as:

(9) $$U_{i}=\sum_{j}{\tilde{p}}_{i,j}\cdot w_{j}$$

where *i* means the *i*-th segment and *j* means the *j*-th segment attribute.

(2) Route resistance

The route attributes include cost of time (*r_m,1_*), fuel consumption (*r_m,2_*), toll fee (*r_m,3_*), the number of traffic lights (*r_m,4_*), the number of intersections (*r_m,5_*) and the number of turns (*r_m,6_*). These attributes cannot be simply calculated from their segments. They are affected by intersections as well. The model of DPRP is as follows:

(10) $$\mathrm{Min}\;V_{m}=\sum_{i\in R_{m}}U_{i}+\left|R_{m}\right|\cdot \sum_{n}{\tilde{r}}_{m,n}w_{n}$$

where *m* means the *m*-th route and *n* means the *n*-th route attribute. The first item on the left side of (10) represents the sum of the road resistance of all segments belong to m. *R_m_* is the set of all segments belonging to *m*. We use *|R_m_|*, i.e., the number of segments in route *m*, to keep the value of segment and route resistance at the same level.

## 3. Proposed Solution Approach

According to DPRP proposed in Section 2, we can recommend a route that satisfies a driver’s preference. However, the attributes are classified, and the value of the route attribute part cannot be calculated when routes are not determined. In a general road network, there are countless routes from origin to destination. If they are all listed, computing their *V_m_* may take too much time and memory, which is unrealistic and unnecessary.

In order to make a DPRP model solvable, we choose routes that meet some most important conditions to reduce unnecessary work. Distance is one of the most important attributes that affect a driver’s choice. In most cases, a route loses its meaning when the distance is too much beyond the shortest route, even if the other attributes of this route are excellent. Moreover, distance is easy to obtain and compare. Therefore, it is used as a condition for initial screening, i.e., the route exceeding a specified distance value is discarded. Here, we use threshold distance (*α*) to control the length of a route. We define the coefficient of the threshold distance, denoted as *α_c_*, as threshold distance (i.e., the longest acceptable distance) divided by the shortest distance. For example, given the shortest distance = 10 km and *α_c_* = 1.3, an acceptable route length is 13 km; i.e., α = 13 km.

Next, we focus on the algorithms to find routes that meet the requirements and give the corresponding time complexity. We propose an integrated algorithm to solve the DPRP model by combining advantages of Algorithms 1 and 2. **Algorithm 1** Removing points and edges that fail the distance requirement  Input: Origin *O*, Destination *D*, Road network *G*, Threshold distance *α*  Output: Set of routes that meet the distance requirementset *A* ← Dijkstra (*G*, *O*, all points out of *O*)set *B* ← Dijkstra (*G*, *D*, all points out of *D*)//sets *A* and *B* just store the distance valuefor all points            if *A_i_* + *B_i_* > *α*                                    delete point *i* in *G/*/*i* is not connected to other point            end ifend forfor all the segment                    if *A_j_* + *L_segment*(*j*, *k*) + *B_k_* > *α*                                    *G*(*j*, *k*) ← inf                    end if                    if *B_j_* + *L_segment*(*j*, *k*) + *A_k_* > *α*                                    *G*(*j*, *k*) ← inf                     end ifend forfind all the routes form *O* to *D* in new road networkoutput routes less than *α*

### 3.1. Road Network Simplification Strategy

The idea of our first algorithm aims to reduce the size of the road network and delete unnecessary points and edges. Once the route passes these points and edges, the distance of the route over *α*, which means that these points and edges should not appear in the alternative routes. By deleting these parts, we can avoid accessing routes that exceed *α*. Its complete procedure is described in Algorithm 1. First, verify points: The shortest distances from each point vi to origin (*O*) and destination (*D*) are calculated by using the Dijkstra algorithm, and are stored in data sets A and B. Each point in A and B is correspondingly added, and the results are compared with *α*. If the result is over *α*, the point is deleted in the road network. Second, verify edges: the length of an edge is added to the distance between the two points on this edge and compared with *α*. If the result is over *α*, the edge is deleted in the road network. Finally, all routes between *O* and *D* are found in the processed road network, and the routes are deleted if their total distance exceeds *α*.

**Theorem** **3.1.** *The asymptotic computational complexity of Algorithm 1 is O(c^I^^-x + 1^)*.

**Proof.**  First of all, we summarize the asymptotic complexity of each step in Algorithm 1 in Table 4.

Based on Table 4, we formulate the computational complexity of Algorithm 1 as:
*T*_1_ = 2O(*I* + |*ν*|log|*ν*|) + O(|*ν*| + 2*I*) + O(*c^I^*^-*x* + 1^) + O(1)
≈O(*c^I^*^-*x* + 1^)
where *c* is the number of connections from an intersection to other intersections that is the maximum in a road network, and *x* is the number of deleted edges. Algorithm 1 reduces the computational burden by reducing the size of a road network, but deleting these points and edges cannot completely avoid routes whose distance exceeds *α*. In Step 17, time is wasted on not meeting the distance requirements for a route. □

### 3.2. Pruning Algorithm

Our pruning algorithm is based on the depth-first search traversal concept. It monitors the distance when accessing a route. Once it finds that the distance exceeds the threshold, it stops visiting the next point and cuts off the routes to be generated. Realized as Algorithm 2, it has the following steps: (1) visit the origin and mark it as being visited; (2) randomly visit the unvisited points connected to the origin and mark as being visited; (3) record the route distance. If the distance is over *α*, go back to the previous point, otherwise visit the next point randomly that links to the current point; (4) update the distance of this route and compare it with the threshold. This process is repeated until the destination is reached. Output this segment, set the destination as being unvisited, and go back to the previous point. Repeat the above process until all routes are found.
**Algorithm 2** Pruning algorithm based on depth-first search for traversal segment of two pointsInput: Origin *O*, Destination *D*, Road network *G*, Threshold distance *α*Output: Set of routes that meet the distance requirementaccess origin *O*, mark *O* as accessedrandom access to the point that is connected to *O* and it is not accessedcalculate route distance *D*if *D* > *α*, go back to last pointrandom access to the point that is connected to last point and it is not accessedif *D* < *α* and this point is not destination, random access to pointrepeat step 3–6until access destination *D*output this route, set *D* to not accessed, go back to last pointrandom access to the point that is connected to last point and it is not accessedrepeat step 3–6until access destination *D* and It doesn’t repeat the previous routesoutput this routerepeat step 3–13, until find all routes

**Theorem** **3.2.** *In the worst-case, the asymptotic computational complexity of Algorithm 2 is O(c^I^ ^+ 1^). In the best-case, its complexity is linear with the number of segments*.

**Proof.**  The computational complexity of Algorithm 2 needs to be discussed in different cases. (1) In the worst-case, all routes do not exceed the threshold, i.e., no segment is cut out. Algorithm 2 is the same to the traversal algorithm with its complexity equal to the traversal algorithm, O(*c^I^* ^+ 1^). (2) In the best-case, Algorithm 2 can cut out most of the routes. Hence, we can quickly find the desired routes. Consequently, its complexity is linear with the number of segments. (3) On average, its performance is better than the traversal algorithm but worse than a linear one. □

### 3.3. Integrated Algorithm

Algorithms 1 and 2 speed up the search for required routes from different aspects. Algorithm 1 reduces the calculation by simplifying the road network and Algorithm 2 removes the invalid sub-routes in a search process. We propose Algorithm 3 that integrates them to improve the performance.**Algorithm 3** Combination of Algorithms 1 and 2Input: Origin ***O***, Destination ***D***, Road network ***G***, Threshold distance ***α***
Output: Set of routes that meet the distance requirement 
$\tilde{G}$← Algorithm 1 (***G***, ***O***, ***D***, ***α***)***A***← Algorithm 2 ($\tilde{G}$, ***O***, ***D***, ***α***)output ***A***

**Theorem** **3.3.** *In the worst-case, the asymptotic computational complexity of Algorithm 3 is O(c^I^^-x + 1^). In the best-case, its complexity is linear with the number of segments*.

**Proof.**  The time complexity of Algorithm 3 is similar to that of Algorithm 2 by noting that Algorithm 2 takes algorithm 1’s output network in Algorithm 3. The difference is that in the worst-case, Algorithm 3’s complexity drops to O(*c^I^*^-*x* + 1^) from Algorithm 2’s.

Algorithm 2 beat Algorithm 1 in most cases to be tested. Algorithm 3’s performance is in general better than Algorithms 1 and 2. Table 5 shows three algorithms’ pros and cons and their time complexity.

Since we use an algorithm to find the shortest and fastest route when we are looking for a route that we want, DPRP can give three results that drivers are interested in after running once, namely, the shortest, fastest and recommended ones considering driver’s preference. □

### 3.4. Large Road Network Resolution Strategy

When DPRP has to deal with route planning with a large road network, it may take much time. DPRP adopts the strategy of zoning in order to solve it. First, when an O–D pair are very far away, it will divide origin and destination into different areas. Then, it determines a route that connects two areas (usually a freeway or expressway) and this route’s origin and destination are O ‘and D’. DPRP recommends routes between O–O ‘and D–D’. Eventually it will give a driver a route that satisfies requirements. For example, from Guangzhou to Shanghai the expressway between two cities will be invoked as a route connecting the two areas.

## 4. Experimental Results and Discussions

In this section, we illustrate DPRP via a simple example and then perform four kinds of experiment to evaluate the proposed methods.

### 4.1. A Simple Example

A simple example is given to introduce how DPRP works. We need to make some assumptions as follows:

(1) Points and edges to describe a road network are used to represent all intersection’s types and road line patterns, respectively.
(2) All alleys, aerodrome roads and port roads are ignored.
(3) Vehicles in a road network are all compact car, occupying a length of 5 m when parked.
(4) On roads where motor vehicles and non-motor vehicles are separated, the number of non-motor vehicles is considered to be zero.

**Figure 1:**
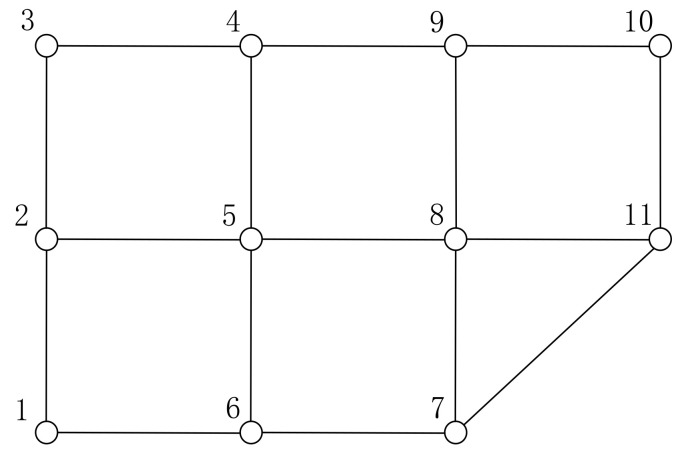
Simple example of a road network.

Figure 1 shows a road network example. The network is an undirected graph consisting of 11 points and 16 edges.

(11) $$\begin{array}{cccccccccccccc} & & p_{1} & p_{2} & p_{3} & p_{4} & p_{5} & p_{6} & p_{7} & p_{8} & p_{9} & p_{10} & & \\ P= & ( & \begin{array}{c} 3\\ 2\\ 3\\ 3\\ 5\\ 2\\ 5\\ 2\\ 3\\ 4\\ 1\\ 4\\ 1\\ 3\\ 5\\ 2 \end{array} & \begin{array}{c} \infty \\ \infty \\ 300\\ \infty \\ \infty \\ 500\\ \infty \\ \infty \\ 900\\ \infty \\ 1000\\ 2000\\ \infty \\ \infty \\ \infty \\ 1500 \end{array} & \begin{array}{c} 1\\ 2\\ 2\\ 4\\ 3\\ 2\\ 3\\ 2\\ 4\\ 3\\ 3\\ 3\\ 2\\ 4\\ 3\\ 2 \end{array} & \begin{array}{c} 3.5\\ 4.0\\ 4.0\\ 4.5\\ 4.0\\ 4.0\\ 4.0\\ 3.5\\ 4.5\\ 4.0\\ 4.0\\ 4.0\\ 4.0\\ 4.5\\ 4.0\\ 4.0 \end{array} & \begin{array}{c} 300\\ 400\\ 300\\ 400\\ 400\\ 300\\ 400\\ 300\\ 400\\ 400\\ 300\\ 500\\ 300\\ 400\\ 400\\ 500 \end{array} & \begin{array}{c} 5\\ 0\\ 5\\ 10\\ 5\\ 10\\ 0\\ 0\\ 15\\ 0\\ 5\\ 0\\ 0\\ 20\\ 5\\ 0 \end{array} & \begin{array}{c} 4/3\\ 6/4\\ 6/3\\ 19/4\\ 12/4\\ 14/3\\ 15/4\\ 9/3\\ 24/4\\ 10/4\\ 7/3\\ 8/5\\ 11/3\\ 36/4\\ 9/4\\ 12/5 \end{array} & \begin{array}{c} 15/3\\ 0\\ 8/3\\ 0\\ 0\\ 4/3\\ 0\\ 0\\ 0\\ 0\\ 0\\ 0\\ 0\\ 0\\ 13/4\\ 17/5 \end{array} & \begin{array}{c} 6\\ 5\\ 8\\ 1\\ 3\\ 4\\ 3\\ 2\\ 3\\ 1\\ 3\\ 1\\ 5\\ 2\\ 5\\ 6 \end{array} & \begin{array}{c} 1\\ 0\\ 1\\ 0\\ 0\\ 1\\ 0\\ 0\\ 0\\ 0\\ 0\\ 0\\ 0\\ 0\\ 1\\ 1 \end{array} & ) & \begin{array}{c} X_{1-2}\\ X_{1-6}\\ X_{2-3}\\ X_{2-5}\\ X_{3-4}\\ X_{4-5}\\ X_{4-9}\\ X_{5-6}\\ X_{5-8}\\ X_{6-7}\\ X_{7-8}\\ X_{7-11}\\ X_{8-9}\\ X_{8-11}\\ X_{9-10}\\ X_{10-11} \end{array} \end{array}$$

where *p_a_* (*a* = 1, 2, …, 10) is a segment attribute. (*b* = 1, 2, …, 10; *c* = 2, 3, …, 11) is the segment number, i.e., a segment from point “*b*” to point “*c*”. ∞ is infinity, meaning that the segment is a straight line segment. We assume that the traffic volume in both directions of a segment is equal in order to simplify our illustration. 

**Figure 2:**
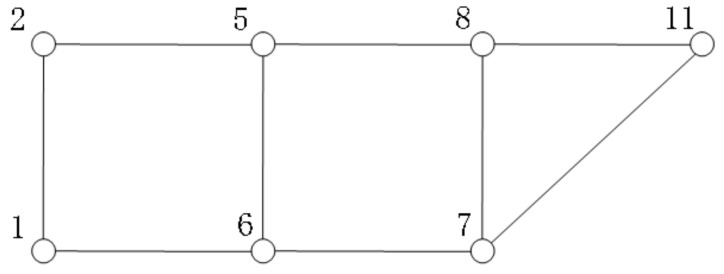
A simplified road network.

In this example, origin is point 1 and destination is point 11 and (10) illustrates the attributes of segments. The shortest route from point 1 to point 11 is “1-6-7-11”, the distance is 1300 m. Setting *α_c_* = 1.2 (i.e., a route exceeding 1.2 times the shortest route is not considered), and using the method in Section 3 to obtain a set of alternative routes. First, according to Algorithm 1, points “3”, “4”, “9”, “10” and edges “2-3”, “3-4”, “4-5”, “4-9”, “8-9”, “9-10” are unavailable. The road network can be simplified as shown in Figure 2. According to Algorithm 2, the routes meeting the constraints can be found. There are four alternative routes, namely, “1-2-5-8-11”, “1-6-5-8-11”, “1-6-7-11”, and “1-6-7-8-11”. Their route attributes are as follows:

(12) $$\begin{array}{ccccccccc} & & r_{1} & r_{2} & r_{3} & r_{4} & r_{5} & r_{6} & \\ R= & ( & \begin{array}{c} 3.45\\ 3.75\\ 2.65\\ 3.45 \end{array} & \begin{array}{c} 1.11\\ 1.18\\ 0.74\\ 1.11 \end{array} & \begin{array}{c} 0\\ 0\\ 15\\ 5 \end{array} & \begin{array}{c} 2\\ 3\\ 1\\ 2 \end{array} & \begin{array}{c} 3\\ 3\\ 2\\ 3 \end{array} & \begin{array}{c} 1\\ 2\\ 1\\ 2 \end{array} & ) \end{array}$$

where *r_a_* (*a* = 1, 2, …, 6) is the route attributes (corresponding to Table 2).

$\tilde{P}$ and $\tilde{R}$ are calculated according to (3)–(6). Table 6 presents a group of weights that are the average values of preferences from 10 men who are 20–30 years old. We use a questionnaire survey to obtain their preference data, i.e.,

(13) $$\tilde{P}=\left(\begin{array}{cccccccccc} 0.17 & 0.00 & 1.00 & 1.00 & 0.00 & 0.25 & 0.00 & 0.95 & 0.71 & 1\\ 0.38 & 0.00 & 0.33 & 0.44 & 0.50 & 0.00 & 0.02 & 0.00 & 0.57 & 0\\ 0.17 & 1.00 & 0.33 & 0.44 & 0.00 & 0.25 & 0.09 & 0.51 & 1.00 & 1\\ 0.17 & 0.00 & 0.00 & 0.00 & 0.50 & 0.50 & 0.45 & 0.00 & 0.00 & 0\\ 0.00 & 0.00 & 0.11 & 0.44 & 0.50 & 0.25 & 0.22 & 0.00 & 0.29 & 0\\ 0.38 & 0.60 & 0.33 & 0.44 & 0.00 & 0.50 & 0.43 & 0.25 & 0.43 & 1\\ 0.00 & 0.00 & 0.11 & 0.44 & 0.50 & 0.00 & 0.32 & 0.00 & 0.29 & 0\\ 0.38 & 0.00 & 0.33 & 1.00 & 0.00 & 0.00 & 0.22 & 0.00 & 0.14 & 0\\ 0.17 & 0.33 & 0.00 & 0.00 & 0.50 & 0.75 & 0.61 & 0.00 & 0.29 & 0\\ 0.06 & 0.00 & 0.11 & 0.44 & 0.50 & 0.00 & 0.15 & 0.00 & 0.00 & 0\\ 1.00 & 0.30 & 0.11 & 0.44 & 0.00 & 0.25 & 0.13 & 0.00 & 0.29 & 0\\ 0.06 & 0.15 & 0.11 & 0.44 & 1.00 & 0.00 & 0.03 & 0.00 & 0.00 & 0\\ 1.00 & 0.00 & 0.33 & 0.44 & 0.00 & 0.00 & 0.30 & 0.00 & 0.57 & 0\\ 0.17 & 0.00 & 0.00 & 0.00 & 0.50 & 1.00 & 1.00 & 0.00 & 0.14 & 0\\ 0.00 & 0.00 & 0.11 & 0.44 & 0.50 & 0.25 & 0.12 & 0.54 & 0.57 & 1\\ 0.38 & 0.20 & 0.33 & 0.44 & 1.00 & 0.00 & 0.14 & 0.57 & 0.71 & 1 \end{array}\right)$$

(14) $$\tilde{R}=\left(\begin{array}{cccccc} 0.73 & 0.85 & 0.00 & 0.50 & 1.00 & 0.00\\ 1.00 & 1.00 & 0.00 & 1.00 & 1.00 & 1.00\\ 0.00 & 0.00 & 1.00 & 0.00 & 0.00 & 0.00\\ 0.73 & 0.85 & 0.33 & 0.50 & 1.00 & 1.00 \end{array}\right)$$

Take one of the routes, i.e., 1-6-7-11, as an example to calculate its road resistance. The calculation process is as follows:
*V*_1-6-7-11_ =(0.38 × 0.36 + 0 × 0.28 + 0.33 × 0.56 + 0.44 × 0.8 + 0.5 × 0.4 + 0 × 0.96 + 0.02 × 0.8 + 0 × 0.68 + 0.57 × 0.82+ 0 × 0.8) + (0.06 × 0. 36 + 0 × 0.28 + 0.11 × 0.56 + 0.44 × 0.8 + 0.5 × 0.4 + 0 × 0.96 + 0.15 × 0.8 + 0 × 0.68 + 0 × 0.82 + 0 × 0.8) + (0.06 × 0.36 + 0.15 × 0.28 + 0.11 × 0.56 + 0.44 × 0.8 + 1 × 0.4 + 0 × 0.96 + 0.03 × 0.8 + 0 × 0.68 +0 × 0.82 + 0 × 0.8) + (0 × 1 + 0 × 0.68 + 1 × 0.2 + 0 × 0.6 + 0 × 0.6 + 0 × 0.74) × 3 = 3.6134

We can use the same method to get other routes’ road resistance (placed in Appendix A). The one with the smallest value is recommended to the driver.

### 4.2. Case Study

In this section, an instance is used to verify the performance of the model in actual situations: route planning in the Coloane-Taipa, Macau. The map of that area can be viewed in Figure 2. The area comprises three bridges connecting the Peninsula of Macao, some parts with dense and regular street patterns, and some sparsely populated area. 

The algorithms devised in this paper are implemented in MATLAB R2015b. We conduct the experiments on a laptop running Microsoft Windows 10 with an Intel/2.5 GHz Core (TM) i5-7300HQ CPU, 8 GB of RAM, and 1 TB of hard disk.

**Figure 3:**
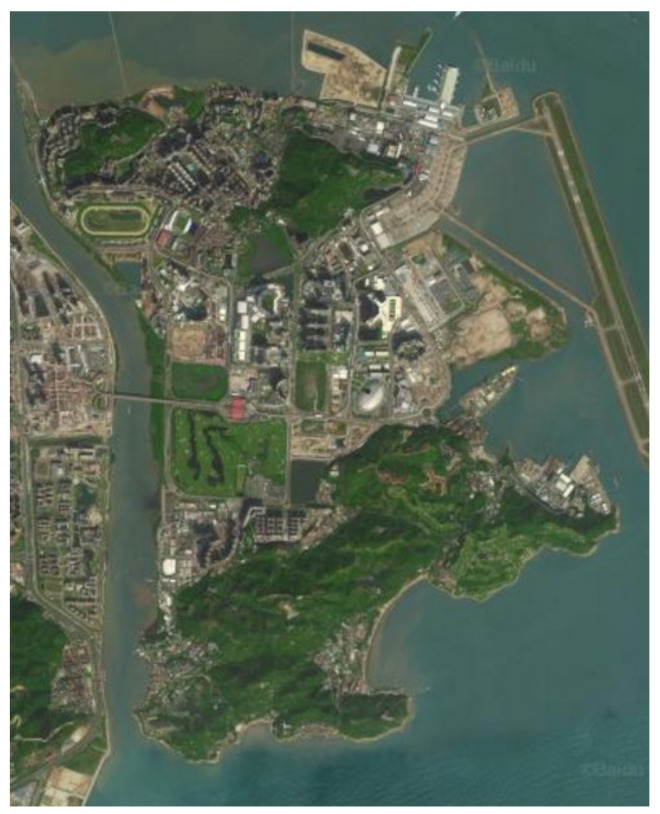
The Coloane-Taipa map.

In order to facilitate the description, the road network is simplified to a point-and-line diagram according to our assumptions, consisting of 82 points and 135 edges (as shown in Figure 3. In order to evaluate our algorithms well, four pairs of O–D are selected for the experiment, which are 1→75, 4→74, 7→82 and 48→61, respectively. They represent routes from northwest to southeast, north to south, northeast to southwest and west to east. Five different *α_c_* are used (*α_c_* = 1.10, 1.15, 1.20, 1.25 and 1.3). The weights are the same as those in Section 4.1. Four different experiments are performed to evaluate the performance of DPRP, as given below.

- Experiment 1: The first experiment is about the comparison of the performance of the three algorithms. Since the main time cost of DPRP is to find the set of alternative routes and the subsequent work is the same, only the time cost of each algorithm is compared.
- Experiment 2: When different thresholds are set, the segment set changes, and the influence of different thresholds on the recommended route is compared.
- Experiment 3: It is about comparing the recommended routes in different traffic situations.
- Experiment 4: The model’s response when an accident occurs.

(1) Algorithm performance

**Figure 4:**
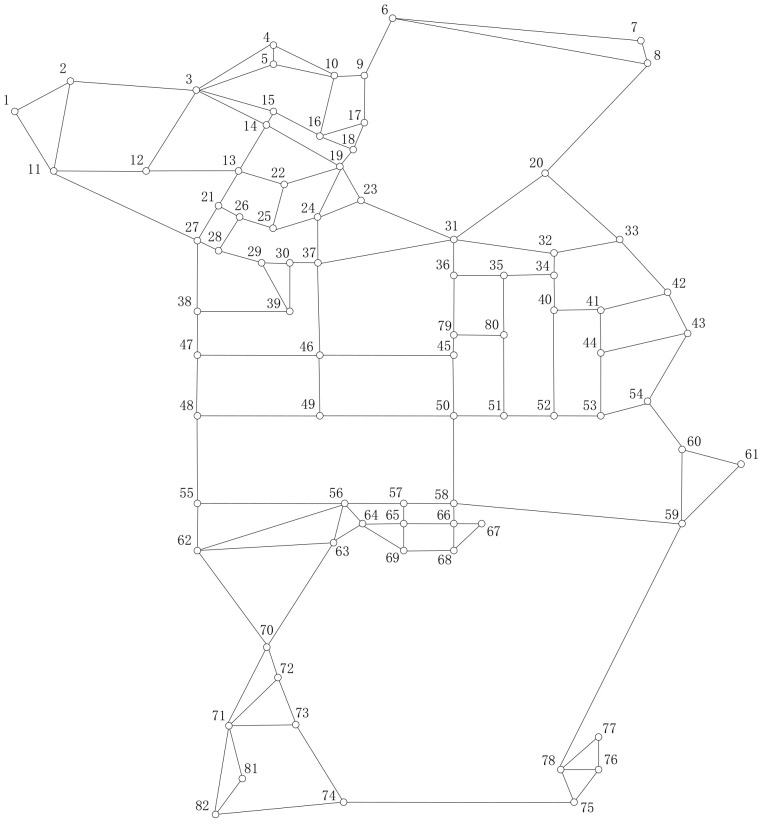
Test network of Coloane-Taipa area.

To evaluate the performance of algorithms in term of the computational efficiency, we compare the K-shortest algorithm and integrated algorithm under four pairs of O–D and different *α_c_* [34,35,36]. The K-shortest algorithm also meets the requirements of DPRP. It can give routes that meet the requirements and calculate the values of route attributes. Since the integrated algorithm combines advantages of Algorithms 1 and 2, we just compare it with the K-shortest algorithm. The results are reported in Figure 4. Here, we can see that as the threshold increases, the time required to find routes that meet the requirements also increases. Because a larger threshold distance means more acceptable routes in a given O–D pair of a road network, more computation is required. Considering the four O–D pairs, the performance of integrated algorithm is better. The number of alternative routes that meet the requirements is shown in Table 7. From it, we can find that when there are many alternative routes, the integrated algorithm spends less time than the K-shortest algorithm.

**Figure 5:**
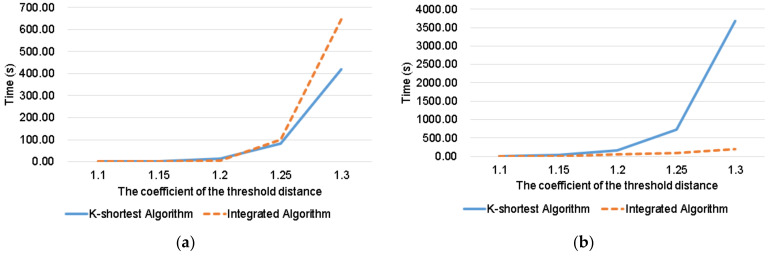

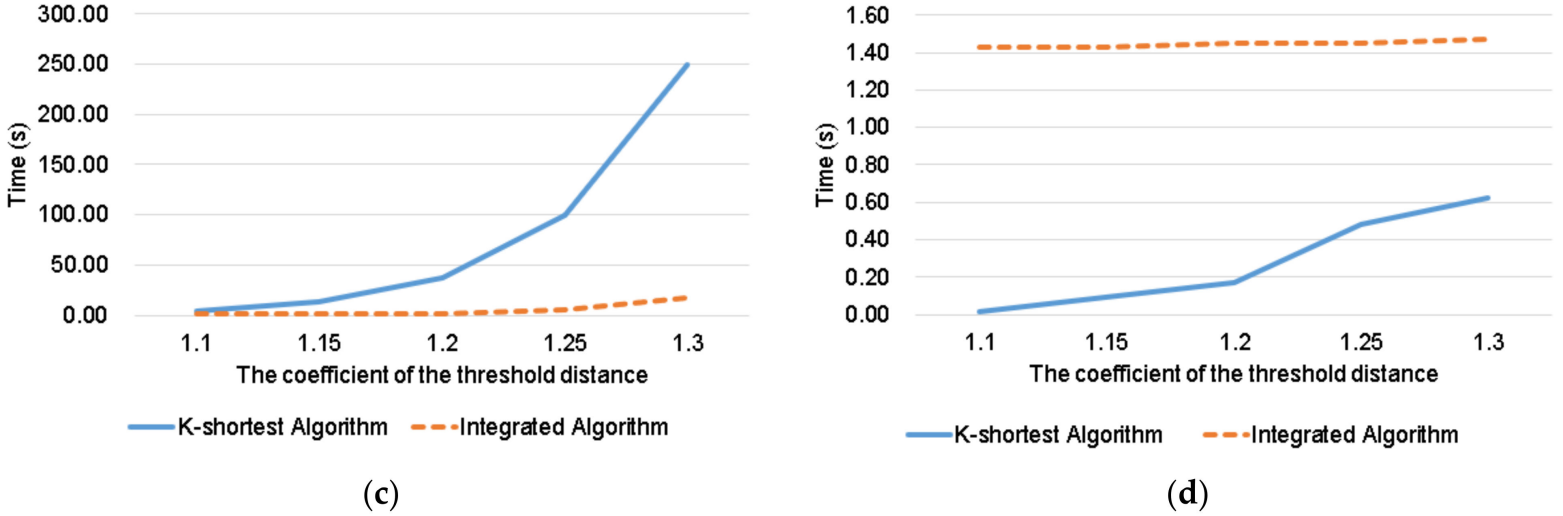
Computational time for the route from (**a**) points 1 to 75. (**b**) points 4 to 74. (**c**) points 7 to 82. (**d**) points 48 to 61.

In Figure 5a, the time cost of K-shortest algorithm is significantly less than the integrated algorithm when *α_c_* = 1.3. In order to verify our conclusion, an additional experiment when *α_c_* = 1.4 is added. The results show that the K-shortest algorithm tales 14,714 s and the integrated algorithm takes 2656 s. The performance of the integrated algorithm is better than the K-shortest algorithm when there are many alternative routes. The K-shortest algorithm needs some time to sort routes, while it is not required for DPRP. In some special cases, the performance of the K-shortest algorithm is better than the integrated algorithm, e.g., when there are only a few points and paths that can be ignored in a road network. However, as the number of routes increases, the performance of the K-shortest algorithm becomes worse.

The performance of the K-shortest algorithm is the worst in Figure 5b, because in 4→74 when α_c_ = 1.3 there are the most alternative routes in all cases we consider. In Figure 5d, due to the small number of routes, the difference in time is not obvious.

(2) Impact of distance threshold on the recommended route

This sub-section presents the influence of different *α_c_* on planning a route. It is determined from the result in Table 8 that a planned route may change with *α_c_*, but the influence is not significant. The main reason is that distance is the limiting condition; while it is also considered as an important attribute by most people. It also affects other attributes such as time and the number of intersections. The results of 1→75 and 4→74 do not change, 7→82 and 48→61 are stable when *α_c_* = 1.25. Due to the importance of the distance attribute, it is very unlikely to obtain a new result even if *α_c_* continues to increase. Moreover, if *α_c_* is too large, the result may not be accepted, which means that a driver needs to travel too much extra distance.

As *α* increases, we obtain more alternative routes to find a recommended route. However, we have to spend more time finding them and the cost usually grows exponentially which can be a challenging issue for a large network. Considering the accuracy of the recommendation and the computational cost, *α_c_* is set to 1.3 in the subsequent experiments.

(1) Impact of different traffic volume on the recommended route

In Table 9, we test DPRP under two traffic volumes. The data used in time bucket 1 is the same as before. Road sections “37-46-49-48”, “50-58-59” and “70-72-73” have greater traffic volume in time bucket 2. We see that DPRP gives different routes due to increased traffic volume on these road sections. Some new routes avoid the segment “70-72-73” in 1→75 and 4→74. In 7→82, the road section is changed from “31-37-46-49-48-55-62-70” to “31-36-79-45-50-58-57-56-63-70”.

(2) Impact of close segments on the recommended route

In urban roads, sometimes a certain segment may be closed or seriously congested due to construction or an accident. It costs extra time to pass through these road sections. After obtaining relevant information, the recommended route should be updated immediately to give feedback to a driver. For the case that there are closed roads in a road network, we calculate a new route by our model and compare it with the previous route, which is provided in Table 10 We assume that the closed segments are 47-48 and 50-58. It is clear that DPRP can provide another optimal route for a driver to avoid the closed segment when it receives the information of the closed segment.

### 4.3. Comparing Existing Approaches

Since the results of route planning based on driver preference are subjective to a certain extent, it is difficult to answer which approach offers the best route in practice. At the same time, the attributes considered in different approaches are different. These approaches cannot be compared directly, but we can compare them in other ways. In this study, we compare the proposed DPRP to a driver-centric route guidance system (DCRGS) [12] and dynamic route search method (DRSM) [19]. The comparison results are shown in Table 11.

It can be observed from Table 11 that the proposed DPRP considers 16 attributes which affect drivers’ choices, while the compared approaches just consider 6-7 attributes. In our opinion, attributes should be considered as comprehensively as possible in a model. If drivers are not interested in some attributes, they can ignore these attributes by setting the weight to “No importance at all” when using the proposed navigation systems. However, if we do not consider comprehensively, a model may not be able to meet the demands of some drivers. 

Comparing to the existing approaches, the proposed DPRP is more friendly to drivers. It gives drivers more choices. With the help of big data, drivers can start to use it directly or after setting their preferences. DCRGS uses the way in which drivers set preferences. However, it needs drivers to set up completely on their own, which is inconvenient to drivers. DRSM does not require drivers to set any preference because it does not consider the impact of weights on its routing results.

The proposed DPRP is experimented with a real road network. The size of the road network is 82 points and 135 edges. DCRGS is tested on a small and simulated road network, so we cannot evaluate their performance in the actual large road network. DRSM is experimented on in a real road network with 7186 points and 22,813 edges. Because it uses a heuristic algorithm to solve the model, its computation time can be accepted in a large road network. However, it does not consider the weight of attributes; it uses the fuzzy-AHP (analytic hierarchy process) method to obtain relevant parameters, which may deliver the results cannot be accepted by drivers. 

## 5. Conclusions

In this paper, a new route navigation model based on driver preference is proposed. Unlike most commercially deployed navigation systems, DPRP considers more attributes that affect a driver’s choice, and combines them to recommend the most suitable routes for a driver. An algorithm is developed to solve DPRP, and four experiments are conducted. Their time cost, the influence of threshold distance, traffic volume, and closed segments on route planning, are adopted to test DPRP performance. Experimental results verify its feasibility and suitability.

This work has some limitations. The quality of results may be different for different drivers and it is difficult to express this quantitatively. The method of reducing the set of solutions is not suitable for very large road networks. As future work, we plan to find a method for evaluating a DPRP model and other models based on driver preferences for such networks. Heuristic algorithms to handle them hierarchically should be developed [37,38,39,40,41,42,43,44,45,46,47].

## Figures and Tables

**Table 1:** Definition of relative parameters.

Symbol	Description
*V_m_*	The value of road resistance of the *m*-th route
*U_i_*	The value of road resistance of the *i*-th segment
*w_j_* and *w_n_*	Weight of the *j*-th and *n*-th attributes, respectively
*p_i,j_*	The value of the *i*-th segment’s *j*-th attribute
*P*	Attribute matrix = [*p_i,j_*]*_I_*_× *J*_
${\overset{ˇ}{p}}_{j}$	The minimum of the *j*-th attribute
${\hat{p}}_{j}$	The maximum of the *j*-th attribute
*q_i,j_*	Ratio value of the *i*-th segment’s *j*-th attribute
${\overset{ˇ}{q}}_{j}$	The minimum ratio value of the *j*-th attribute
${\hat{q}}_{j}$	The maximum ratio value of the *j*-th attribute
${\tilde{p}}_{ij}$	Value normalized to [0, 1] of the *i*-th segment’s *j*-th attribute
$\tilde{P}$	Attribute matrix = [${\tilde{p}}_{ij}$] *_I_*_× *J*_
*R_m,n_*	The value of the *m*-th route’s *n*-th attribute
*R*	Attribute matrix = [*r_m,n_*] *_M_*_× *N*_
${\overset{ˇ}{r}}_{n}$	The minimum ratio value of the *n*-th attribute
${\hat{r}}_{n}$	The maximum ratio value of the *n*-th attribute
λ _*m,n*_	Ratio value of the m-th route’s *n*-th attribute
λ ˇn	The minimum ratio value of the *n*-th attribute
${\hat{\lambda }}_{n}$	The maximum ratio value of the *n*-th attribute
${\tilde{r}}_{m,n}$	Value normalized to [0, 1] of the *m*-th route’s *n*-th attribute
$\tilde{R}$	Attribute matrix = [${\tilde{r}}_{ij}$] *_M_*_× *N*_
*R_m_*	The set of segments of the *m*-th route
$\left|R_{m}\right|$	The number of segments of the *m*-th route
*I*	The number of segments
*J*	The number of segment attributes
*M*	The number of routes
*N*	The number of route attributes
*α*	Threshold distance
*α_c_*	The coefficient of the threshold distance
*L_i_*	The length of segment *i*

**Table 2:** Definitions of attributes.

Symbol	Description	Type
*p_i,1_*	Scenery	+
*p_i,2_*	Radius of curvature	+
*p_i,3_*	The number of lanes	+
*p_i,4_*	Lane width	+
*p_i,5_*	Distance	-
*p_i,6_*	Congestion	-
*p_i,7_*	Traffic flow	-
*p_i,8_*	The number of pedestrians and bicycles	-
*p_i,9_*	Congestion rate	-
*p_i,10_*	The separation of motor vehicles and non-motor vehicles	-
*r_m,1_*	Cost of time	-
*r_m,2_*	Fuel consumption	-
*r_m,3_*	Toll fee	-
*r_m,4_*	The number of traffic lights	-
*r_m,5_*	The number of intersections	-
*r_m,6_*	The number of turns	-

+: Positive ones, -: Negative ones. *i* means the *i*-th segment, *m* means the *m*-th route.

**Table 3:** Selection interface allowing drivers to select their preferences.

	Most Important	Very Important	Important	Slightly Important	Least Important	No Importance at All	Default
Scenery	○	○	○	○	○	○	○

**Table 4:** Complexity Analysis of Algorithm 1.

Operation	Complexity
set *A* ← Dijkstra (*G*, *O*, all points out of *O*)	O(*I* + |v|log|v|)
set *B* ← Dijkstra (*G*, *D*, all points out of *D*)	O(*I* + |v|log|v|)
//sets *A* and *B* just store the distance	-
for all points	O(*ν*)
if *A_i_* + *B_i_* > *α*	-
delete point *i* in *G*	O(1)
end if	-
end for	-
for all the segment	O(*I*)
if *A_j_* + *L_segment*(*j*, *k*) + *B_k_* > *α*	-
*G*(*j*, *k*) ← inf	O(1)
end if	-
if *B_j_* + *L_segment*(*j*, *k*) + *A_k_* > *α*	-
*G*(*j*, *k*) ← inf	O(1)
end if	-
end for	-
find all the routes form *O* to *D*	O(*c^I^*^-*x* + 1^)
output routes less than *α*	O(1)

**Table 5:** Pros and Cons of Three Algorithms.

Algorithms	Pros and Cons	Time Complexity
Algorithm 1	It can effectively simplify a road network and make easier to find routes, but it wastes time on invalid routes.	O(*c^I^*^-*x* + 1^)
Algorithm 2	It can prevent the production of non-conforming route in time, but its performance is affected by a road network.	Worst-case, O(*c^I^* ^+ 1^); Best-case, O(*I*)
Algorithm 3	It integrates the advantages of algorithm 1 and 2 in different aspects. It has better performance.	Worst-case, O(*c^I^*^-*x* + 1^); Best-case, O(*I*)

**Table 6:** The weights of attributes.

Attribute	Weight
Scenery	Not important (0.36)
Radius of curvature	Do not consider it (0.28)
The number of lanes	Important (0.56)
Lane width	Important (0.8)
Distance	Not important (0.4)
Congestion	Most important (0.96)
Traffic flow	Most important (0.8)
The number of pedestrians and bicycles	Very Important (0.68)
Rate of congestion	Very Important (0.82)
Separating motor vehicles and non-motor vehicles	Most important (0.8)
Cost of time	Most important (1.00)
Fuel consumption	Important (0.68)
Toll fee	Do not consider it (0.2)
The number of traffic lights	Important (0.6)
The number of intersections	Important (0.8)
The number of turns	Very Important (0.74)

**Table 7:** The number of alternative routes.

*O-D*\*α_c_*	1.10	1.15	1.20	1.25	1.30
1→75	16	50	233	1132	4613
4→74	132	652	2437	7760	21,313
7→82	78	311	893	2101	4636
48→61	1	2	4	9	14

**Table 8:** Planned routes in different situations.

*O-D*	*α_c_*	Planned Route
1→75	1.10	**1**→11→27→38→47→48→55→62→70→72→73→74→**75**
	1.15	**1**→11→27→38→47→48→55→62→70→72→73→74→**75**
	1.20	**1**→11→27→38→47→48→55→62→70→72→73→74→**75**
	1.25	**1**→11→27→38→47→48→55→62→70→72→73→74→**75**
	1.30	**1**→11→27→38→47→48→55→62→70→72→73→74→**75**
4→74	1.10	**4**→3→14→13→21→27→38→47→48→55→62→70→72→73→**74**
	1.15	**4**→3→14→13→21→27→38→47→48→55→62→70→72→73→**74**
	1.20	**4**→3→14→13→21→27→38→47→48→55→62→70→72→73→**74**
	1.25	**4**→3→14→13→21→27→38→47→48→55→62→70→72→73→**74**
	1.30	**4**→3→14→13→21→27→38→47→48→55→62→70→72→73→**74**
7→82	1.10	**7**→8→20→31→36→79→45→50→58→57→56→63→70→71→**82**
	1.15	**7**→8→20→31→36→79→45→50→58→57→56→63→70→71→**82**
	1.20	**7**→8→20→31→37→46→49→48→55→62→70→71→**82**
	1.25	**7**→8→20→31→37→46→49→48→55→62→70→71→**82**
	1.30	**7**→8→20→31→37→46→49→48→55→62→70→71→**82**
48→61	1.10	**48**→49→50→51→52→53→54→60→**61**
	1.15	**48**→55→56→57→58→59→**61**
	1.20	**48**→49→50→58→59→**61**
	1.25	**48**→49→50→58→59→**61**
	1.30	**48**→49→50→58→59→**61**

**Table 9:** Planned routes according to different traffic volumes.

*O-D*	Planned Route	
	Time Bucket 1	Time Bucket 2
1→75	**1**→11→27→38→47→48→55→62→70→72→73→74→**75**	**1**→11→27→38→47→48→55→62→70→71→73→74→**75**
4→74	**4**→3→14→13→21→27→38→47→48→55→62→70→72→73→**74**	**4**→3→14→13→21→27→38→47→48→55→62→70→71→73→**74**
7→82	**7**→8→20→31→37→46→49→48→55→ 62→70→71→**82**	**7**→8→20→31→36→79→45→50→58→57→56→63→70→71→**82**
48→61	**48**→49→50→58→59→**61**	**48**→49→50→58→59→**61**

**Table 10:** Planned Routes Given Some Close Segments.

*O-D*	Planned Routes	
	Time Bucket 1	Time Bucket 2 (Close Segments)
1→75	**1**→11→27→38→47→48→55→62→70→72→73→74→**75**	**1**→11→27→38→47→46→49→48→55→62→73→74→**75**
4→74	**4**→3→14→13→21→27→38→47→48→55→62→70→72→73→**74**	**4**→3→14→19→24→37→46→49→48→55→62→70→72→73→**74**
7→82	**7**→8→20→31→37→46→49→48→55→62→70→71→**82**	**7**→8→20→31→37→46→49→48→55→62→70→71→**82**
48→61	**48**→49→50→58→59→**61**	**48**→55→56→57→58→59→**61**

**Table 11:** Comparison of different approaches.

Method	Number of Attributes	Network Size	Actual Network	Computing Weight	Eliminating Errors	Ease for Drivers
DPRP	16	82 Nodes and 135 Links	Yes	Auto-acquisition and driver setting	Big data	High
DCRGS	7	4 × 4 grid in NS-3	No	Driver setting	-	Low
DRSM	6	7186 Nodes and 22,813 Links	Yes	Not needed	Fuzzy-AHP	High

**Figure A1:**
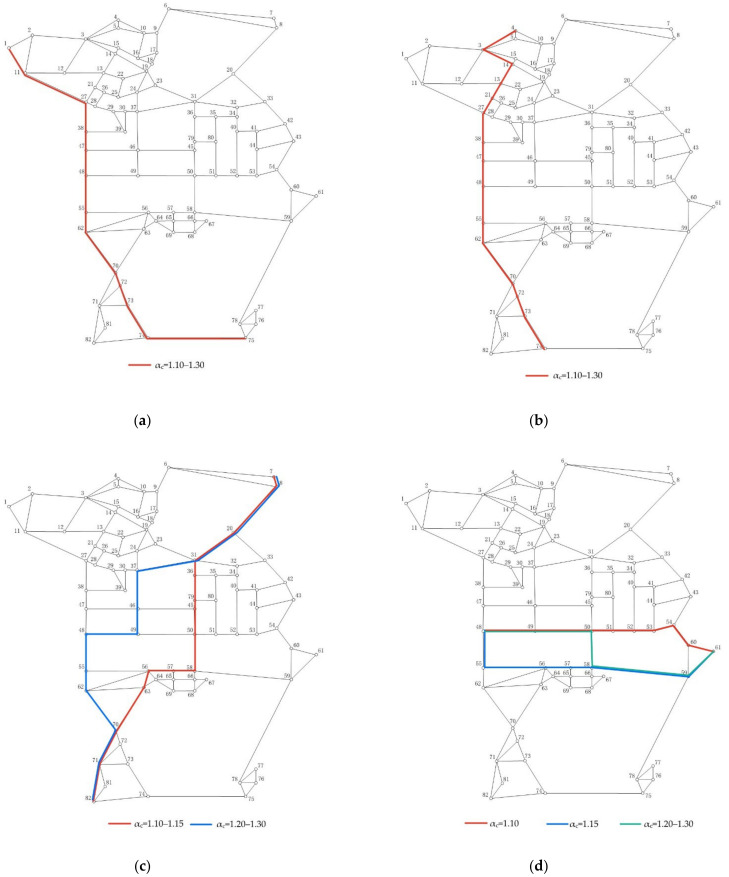
(**a**) The route from points 1 to 75. (**b**) The route from points 4 to 74. (**c**) The route from points 7 to 82. (**d**) The route from points 48 to 61.

**Figure A2:**
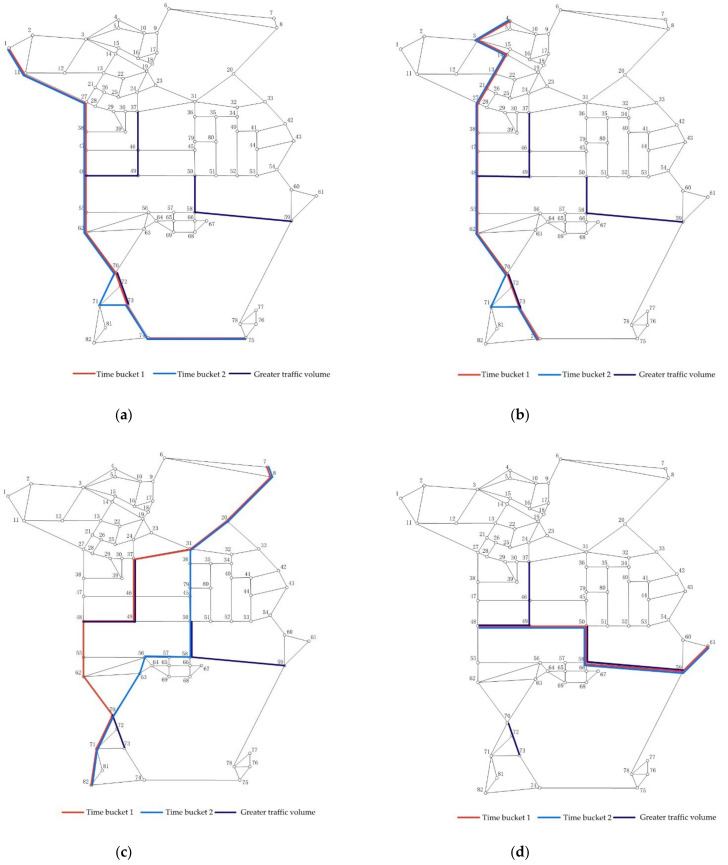
Under different traffic volume, (**a**) The route from points 1 to 75. (**b**) The route from points 4 to 74. (**c**) The route from points 7 to 82. (**d**) The route from points 48 to 61.

**Figure A3:**
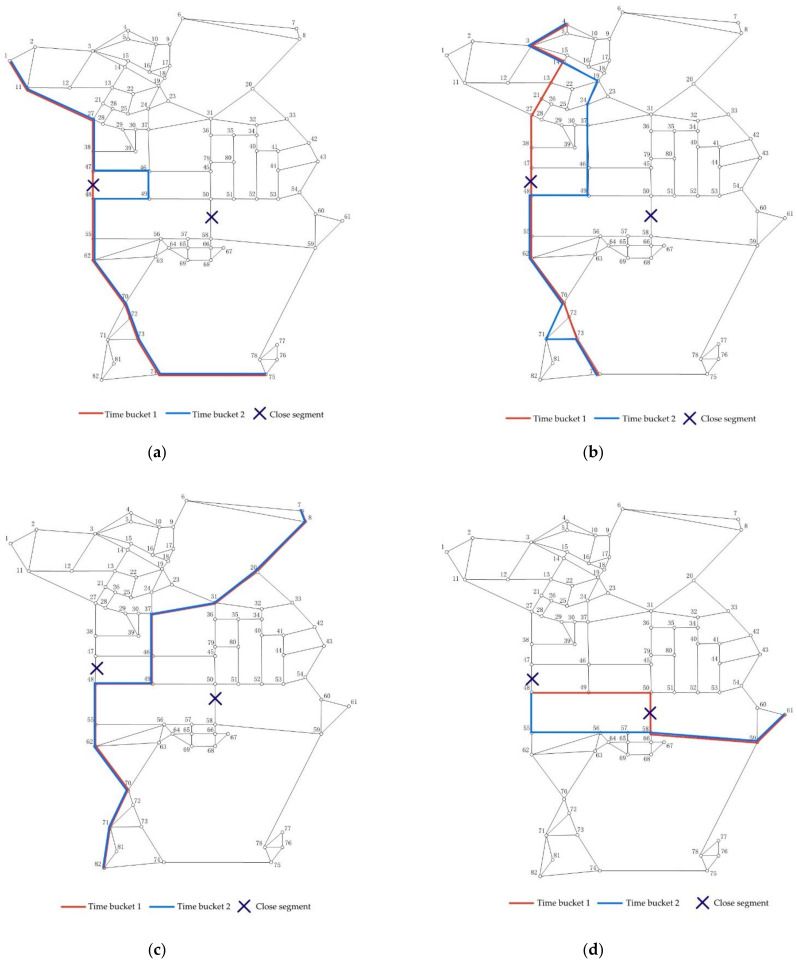
Given some close segments, (**a**) The route from points 1 to 75. (**b**) The route from points 4 to 74. (**c**) The route from points 7 to 82. (**d**) The route from points 48 to 61.

## Data Availability

Data available in a publicly accessible repository.

## Appendix A

1. The value of alternative routes in Section 4.2.

V_1-2-5-8-11_ =
(0.17 × 0.36 + 0 × 0.28 + 1 × 0.56 + 1 × 0.8 + 0 × 0.4 + 0.25 × 0.96 + 0 × 0.8 + 0.95 × 0.68 + 0.71
× 0.82 + 1 × 0.8) +
(0.17 × 0. 36 + 0 × 0.28 + 0 × 0.56 + 0 × 0.8 + 0.5 × 0.4 + 0.5 × 0.96 + 0.45 × 0.8 + 0 × 0.68 + 0 ×
0.82 + 0 × 0.8) +
(0.17 × 0.36 + 0.33 × 0.28 + 0 × 0.56 + 0×0.8 + 0.5×0.4 + 0.75 × 0.96 + 0.61 × 0.8 + 0 × 0.68 + 0.29
× 0.82 + 0 × 0.8) +
(0.17 × 0.36 + 00 × 0.28 + 0 × 0.56 + 0 × 0.8 + 0.5 × 0.4 + 1 × 0.96 + 1 × 0.8 + 0 × 0.68 + 0.14 × 0.82
+ 0 × 0.8) +
(0.73 × 1 + 0.85 × 0.68 + 0 × 0.2 + 0.5 × 0.6 + 1 × 0.6 + 0 × 0.74) × 4 = 17.558

V_1-6-5-8-11_ =
(0.38 × 0.36 + 0 × 0.28 + 0.33 × 0.56 + 0.44 × 0.8 + 0.5 × 0.4 + 0 × 0.96 + 0.02 × 0.8 + 0 × 0.68 +
0.57 × 0.82 + 0 × 0.8) +
(0.38 × 0. 36 + 0 × 0.28 + 0.33 × 0.56 + 1 × 0.8 + 0 × 0.4 + 0 × 0.96 + 0.22 × 0.8 + 0 × 0.68 + 0.14 ×
0.82 + 0 × 0.8) +
(0.17 × 0.36 + 0.33 × 0.28 + 0 × 0.56 + 0 × 0.8 + 0.5 × 0.4 + 0.75 × 0.96 + 0.61 × 0.8 + 0 × 0.68 + 0
× 0.29 + 0 × 0.8) +
(0.17 × 0.36 + 00 × 0.28 + 0 × 0.56 + 0 × 0.8 + 0.5 × 0.4 + 1 × 0.96 + 1 × 0.8 + 0 × 0.68 + 0.14 × 0.
+ 0 × 0.8) +
(1 × 1 + 1 × 0.68 + 0 × 0.2 + 1 × 0.6 + 1 × 0.6 + 1 × 0.74) × 3 = 20.947

V_1-6-7-8-11_ =
(0.38 × 0.36 + 0 × 0.28 + 0.33 × 0.56 + 0.44 × 0.8 + 0.5 × 0.4 + 0 × 0.96 + 0.02 × 0.8 + 0 × 0.68 +
0.57 × 0.82 + 0 × 0.8) +
(0.06 × 0. 36 + 0 × 0.28 + 0.11 × 0.56 + 0.44 × 0.8 + 0.5 × 0.4 + 0 × 0.96 + 0.15 × 0.8 + 0 × 0.68 + 0
× 0.82 + 0 × 0.8) +
(1 × 0.36 + 0.3 × 0.28 + 0.11 × 0.56 + 0.44 × 0.8 + 0 × 0.4 + 0.25 × 0.96 + 0.13 × 0.8 + 0 × 0.68 + 0
× 0.29 + 0 × 0.8) +
(0.17 × 0.36 + 00 × 0.28 + 0 × 0.56 + 0 × 0.8 + 0.5 × 0.4 + 1 × 0.96 + 1 × 0.8 + 0 × 0.68+0.14 ×
0.82 + 0 × 0.8) +
(0.73 × 1 + 0.85 × 0.68 + 0.33 × 0.2 + 0.5 × 0.6 + 1 × 0.6 + 1 × 0.74) × 3 = 17.5058

2. The road-maps corresponding to the data in Table 8, Table 9 and Table 10.
